# End-of-life care for immigrants in Germany. An epidemiological appraisal of Berlin

**DOI:** 10.1371/journal.pone.0182033

**Published:** 2017-08-01

**Authors:** Antje Henke, Peter Thuss-Patience, Asita Behzadi, Oliver Henke

**Affiliations:** 1 Department of Hematology and Oncology, Division of Palliative Medicine, Virchow Campus, Charité University Hospital, Berlin, Germany; 2 Berlin School of Public Health, Charité University Hospital, Berlin, Germany; TNO, NETHERLANDS

## Abstract

**Background:**

Since the late 1950’s, a steadily increasing immigrant population in Germany is resulting in a subpopulation of aging immigrants. The German health care system needs to adjust its services—linguistically, culturally, and medically–for this subpopulation of patients. Immigrants make up over 20% of the population in Germany, yet the majority receive inadequate medical care. As many of the labor immigrants of the 1960s and 1970s are in need of hospice and palliative care (HPC), little is known about this specialized care for immigrants. This epidemiological study presents utilization of HPC facilities in Berlin with a focus on different immigrant groups.

**Methods:**

A validated questionnaire was used to collect data from patients at 34 HPC institutions in Berlin over 20 months. All newly admitted patients were recruited. Anonymized data were coded and analyzed by using SPSS and compared with the population statistics of Berlin.

**Results:**

4118 questionnaires were completed and included in the analysis. At 11.4% the proportion of immigrants accessing HPC was significantly (p<0,001) below their proportion in the general Berlin population. This difference was especially seen in the age groups of 51–60 (21.46% immigrants in Berlin population, 17.7% immigrants in HPC population) and 61–70 years (16,9% vs. 13,1%). The largest ethnic groups are Turks, Russians, and Poles, with a different weighting than in the general population: Turkish immigrants were 24% of all Berlin immigrants, but only 13.6% of the study immigrant population (OR: 0.23, 95%CI: 0.18–0.29, p<0.001). Russian and Polish immigrants account for 5.6% and 9.2% in the population, but 11.5% and 24.8% in the study population respectively (Russian: OR 0.88, 95%CI: 0.66–1.16; Polish: OR 1.17, 95%CI: 0.97–1.42). Palliative care wards (PC) were used most often (16.7% immigrants of all PC patients); outpatient hospice services were used least often by immigrants (11.4%). Median age at first admission to HPC was younger in immigrants than non-immigrants: 61–70 vs. 71–80, p = 0.03.

**Conclusions:**

Immigrants are underrepresented in Berlin´s HPC and immigrants on average make use of care at a younger age than non-immigrants. In this regard, Turkish immigrants in particular have the poorest utilization of HPC. These results should prompt research on Turkish immigrants, regarding access barriers, since they represent the largest immigrant group. This may be due to a lack of cultural sensitivity of the care-providers and a lack of knowledge about HPC among immigrants. In the comparison of the kinds of institutions, immigrants are less likely to access outpatient hospice services compared to PC. Apparently, PC appear to be a smaller hurdle for utilization. These results show a non-existent, but oft-cited “healthy immigrant effect” of the first generation of work immigrants, now entering old age. These findings correspond with studies suggesting increased health concerns in immigrants. Focused research is needed to promote efforts in providing adequate and fair access to HPC for all people in Berlin.

## Introduction

The demographic shift and increase in refugees immigrating to Germany present new challenges to the healthcare system. There is an urgent necessity for the providers of healthcare services to adapt themselves, linguistically, culturally, and medically, to new ethnic groups [1,2]. Already today, immigrants in Germany no longer represent numerically marginal groups. In 2013, there were over 16.5 million immigrants living in Germany [3], which was one-fifth of the entire population [4]. Hospice and Palliative Care (HPC) represents an area of immigrant’s healthcare that is mostly neglected. There are only a few scientific studies addressing this topic in Germany [5,6]. Disparities are known to exist in other areas of healthcare, manifested as poor quality of care [7,8,9]. Studies from other Western industrialized countries describe these disparities in HPC, which are related to communication, pain treatment, diagnostic tests, and the link or access to hospice and palliative care, among others [10,11,12,13]. In particular, the so-called labor immigrants of the 1950s to 1970s are coming to an age when they need specialized care. Contrary to expectations, most of them will remain in Germany for the final stage of their life and the number of older immigrants will continue to rise in the coming years [14]. It is estimated that 15% of all people over age 65 will be immigrants in the year 2030 [15]. Additionally, immigrants as a group often have greater health risks due to their employment history and the psychosocial stress from migration itself [15]. Thus, there is a major need for valid data about end-of-life HPC provision for immigrants.

Epidemiological data on provision of HPC services among immigrants in Germany is lacking and therefore needed to understand the current situation [6]. This would enable the under-provided groups of immigrants to be identified and design targeted solutions.

Therefore, the current work compiles the results of a representative survey of the immigration status of all patients cared for in hospices and palliative care in the City of Berlin. Age groups, gender and immigrant groups are identified. Furthermore, the utilization of the different kinds of institutions by immigrants was investigated.

The frequency of utilization of HPC services among immigrants vs. native Germans, within each of various defined age ranges, were compared.

## Methods

### Study design

For the data collection about the utilization of HPC services by immigrants, a descriptive, exploratory research design, in the form of a full-coverage quantitative cross-sectional study was chosen. This epidemiological study was part of a research project “Hospice and Palliative Care of Immigrants in Berlin”, which also qualitatively investigated viewpoints and expectations of the hospice attendants and the immigrants receiving care [16].

Data collection began in January 2014 and was carried out over 20 months in 34 HPC institutions in Berlin. This included 18 outpatient hospice services, 11 inpatient hospices, and 5 palliative care wards. All newly admitted patients were asked, by means of a questionnaire, about their immigration status, and demographic information.

### Ethics

The study was approved by the local ethics commission and strictly taking into consideration the Declaration of Helsinki of the World Medical Association.

#### Questionnaire

**Fig 1:**
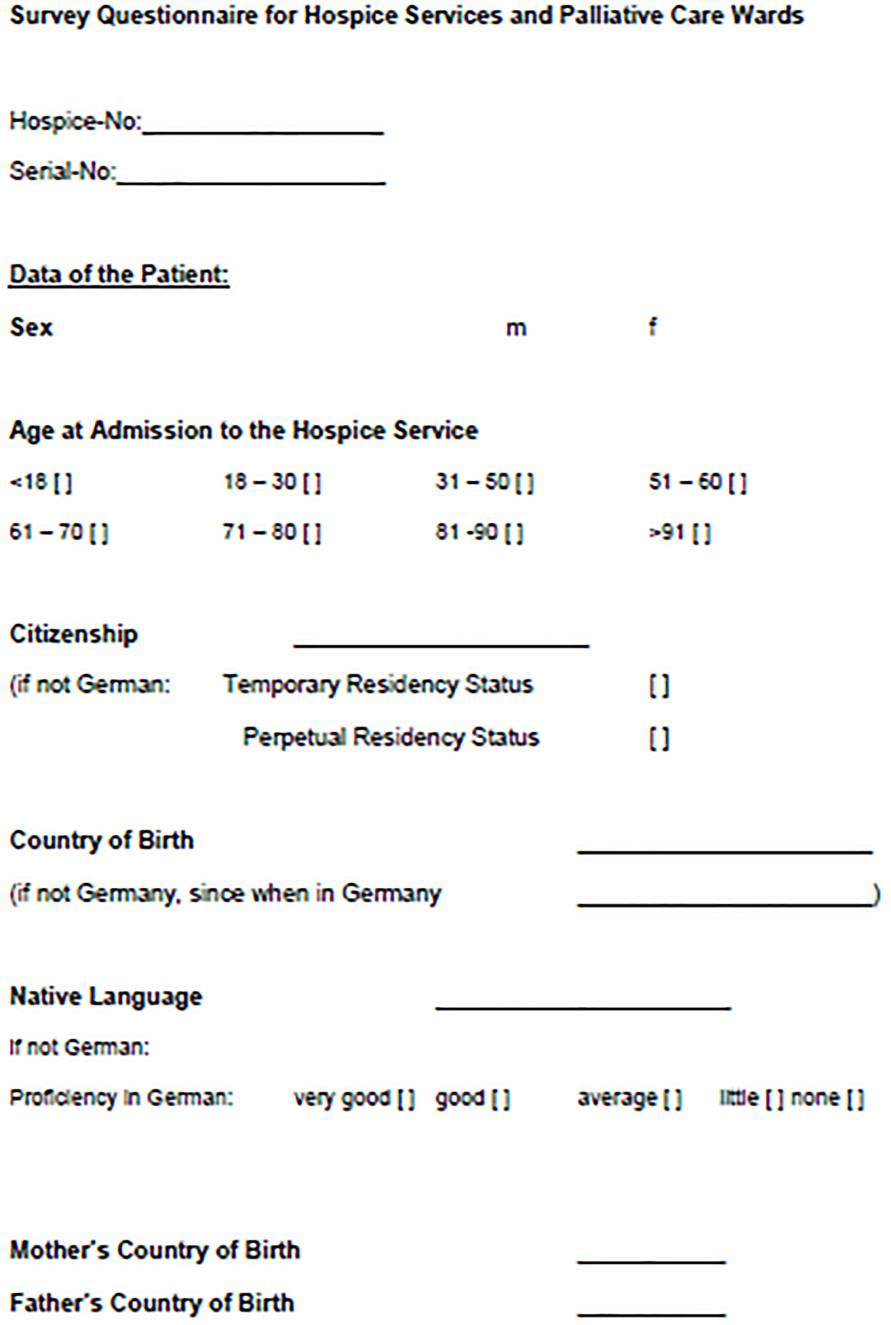
Questionnaire: “Basic Set of Indicators for Mapping Migrant Status”.

Data collection took place by means of a 10-item questionnaire (Fig 1), based on the “Basic Set of Indicators for Mapping Migrant Status”, which was validated and published by Schenk and colleagues in 2006 [17]. The problem for systematically recording the immigration background is its definition. The international literature is often inconsistent and semantically confused [18]. To obtain comparability with the official Berlin population statistics, the authoritative definition was applied: “everyone who migrated into the current territory of the Federal Republic of Germany after 1949, all non-citizens born in Germany, and Germans born in Germany with at least one parent who immigrated into Germany or was born in Germany as a non-citizen” [4]. Furthermore, the questionnaire also included items on the respondent’s sex and age group (Fig 1). The response rate was assessed by comparison of the numbers of questionnaires and effective numbers of patients in random samples of the institutions participating.

### Response rate

34 out of 39 HPC institutions participate in this survey, leading to a coverage of 87,2% of all Berlin HPC institutions (90% of outpatient hospices, 92% of inpatient hospices and 71% of Palliative Care wards). Reasons for non-participation were “shortage of time and staff”.

### Data analysis

Data analysis was performed with SPSS (Statistical Package for the Social Sciences), Win Version 22.0. Data were stratified by age groups, sex and relative frequencies in the partial study samples of the three kinds of institutions. Furthermore, the portion of immigrants for each kind of institutions were analyzed.

In order to determine if the data collected from the Berlin HPCs actually reflected the distribution of immigrants in the population of Berlin, data from the Statistical Office of the State of Berlin-Brandenburg from the year 2014 were compared to the data collected by the present study. Odds Ratio (OR) were calculated by using Chi Square test to indicate comparisons of the statistical portion of immigrants vs. non-immigrants for the whole study sample and for the three biggest groups of immigrants, stratified to age groups and the three kinds of institutions.

## Results

### Gender, age and institutions

A total of 4118 questionnaires were completed and evaluated (1653 from outpatient hospice services, 1378 from inpatient hospices, and 1087 from palliative care wards).

There were more women (56.8%) than men (43.2%) in the sample. The median and mode values for the age group for the entire study sample were both in the age group of 71–80 years old, with, on average, younger patients in the palliative care wards and older patients in the outpatient hospice services (Table 1).

**Table 1:** Sociodemographic data for the entire study sample and of the different kind of institutions.

		outpatient hospice services	inpatient hospices	palliative care wards	Total
	Entire sample	n = 1653	n = 1378	n = 1087	n = 4118
	Immigrants	n = 176	n = 125	n = 170	n = 471
median age group	Entire sample	71–80	71–80	61–70	**71–80**
	Immigrants	61–70	71–80	61–70	**61–70**
Female in %	Entire sample	62.9	53.8	51.5	**56.8**
	Immigrants	56.7	56.7	46.7	**52,0**
**Immigrants in %**		**10.6**	**9.7**	**15.6**	**11.4**
Polish immigrants in %	of entire sample	2.5	2.6	3.6	**2.8**
	of all immigrants	23.9	28.8	22.9	**24.8**
Russian immigrants in %	of entire sample	1.3	0.9	1.9	**1.3**
	of all immigrants	11.9	9.6	12.4	**11.5**
Turkish immigrants in %	of entire sample	0.9	1.2	3.0	**1.6**
	of all immigrants	8.0	13.6	19.4	**13.6**

### Immigrants

Of the total study population, 471 (11,4%) patients were immigrants, 3558 (86,4%) were not immigrants, and 89 (2,2%) remained indeterminate due to missing data answers. The immigrants were distributed among the types of institutions as follows: 176 (10,6%) were in outpatient hospices, 125 (9,1%) were in inpatient hospices, and 170 (15,6%) were in palliative care wards; thus the proportion of immigrants is highest in palliative care wards. The proportion of immigrants declines with rising age groups: 31.5% of immigrants are 31–50 year of age, while only 9.7% are 71–80 years of age (Table 1).

#### Comparison with the population of Berlin

In 2014, 3,443,100 people lived in Berlin, of whom 908,500 (26%) had an immigration background. There is a greater proportion of immigrants in the younger age groups than in the older age groups. While 26% of the Berliners with an immigration background are younger than 18 years, the group with the highest proportion is the 31–50 year with 32.5%. Only 6.5% of immigrants are in the age group of 51–60 years and only 0.6% of 81–90 year of age. The largest immigrant group in Berlin are the Turkish, with a proportion of 6.3% of the total population and 24% of all immigrants, followed by Polish immigrants at 2.4% of the general population (and 9.2% of all immigrants) and Russian immigrants at 1.5% of the general population (and 5.6% of all immigrants).

These three groups are the most common groups in the present study (Table 1), though with different weightings. Turkish immigrants occur three times less in the HPC institutions than in the Berlin general population, while Russian and Polish immigrants are well represented according to their proportion in the general population of Berlin. Table 2 (Table 2) presents the corresponding odds ratio´s (OR) and p-value for the total numbers of immigrants and the largest age groups of the study population.

**Table 2:** Comparison of the largest immigrant groups in the Berlin population and the study population, stratified to the largest age groups. Reference group for all OR´s are their portions in the Berlin population.

	Berlin population	Study population	Odds Ratio & Confidence Interval	p-value
**All** Age group 51–60 Age group 61–70 Age group 71–90	n = 3,443,100	n = 4,118		
	n = 467,600	n = 492		
	n = 350,700	n = 785		
	n = 449,100	n = 2210		
**Immigrants**	n = 908,500	n = 471	0.36 (0.33–0.39)	**<0.0001**
Age group 51–60	n = 100,900	n = 87	0.78 (0.62–0.99)	**0.0356**
Age group 61–70	n = 59,300	n = 103	0.74 (0.60–0.92)	**0.0046**
Age group 71–90	n = 25,500	n = 178	1.46 (1.25–1,71)	**<0.0001**
**Turkish Immigrants** Age group 51–60 Age group 61–70 Age group 71–90	n = 218,630	n = 64	0.23 (0.18–0.30)	**<0.0001**
	n = 19,920	n = 11	0.51 (0.27–0.96)	**0.0261**
	n = 14,630	n = 14	0.42 (0.24–0.72)	**0.0008**
	n = 8,920	n = 26	0.59 (0.39–0.88)	**0.0062**
**Polish Immigrants**	n = 83,730	n = 117	1.17 (0.97–1.42)	0.0879
Age group 51–60	n = 16,650	n = 18	1.03 (0.62–1.68)	0.9067
Age group 61–70	n = 7,920	n = 26	1.48 (0.98–2.23)	0.0467
Age group 71–90	n = 3,300	n = 53	3.36 (2.53–4.45)	**<0.0001**
**Russian Immigrants**	n = 51,190	n = 54	0.88 (0.67–1.16)	0.3518
Age group 51–60	n = 5,620	n = 7	1.19 (0.52–2.58)	0.6528
Age group 61–70	n = 4,810	n = 5	0.46 (0.17–1.15)	0.0765
Age group 71–90	n = 2,710	n = 35	2.67 (1.88–3.78)	**<0.0001**

**Fig 2:**
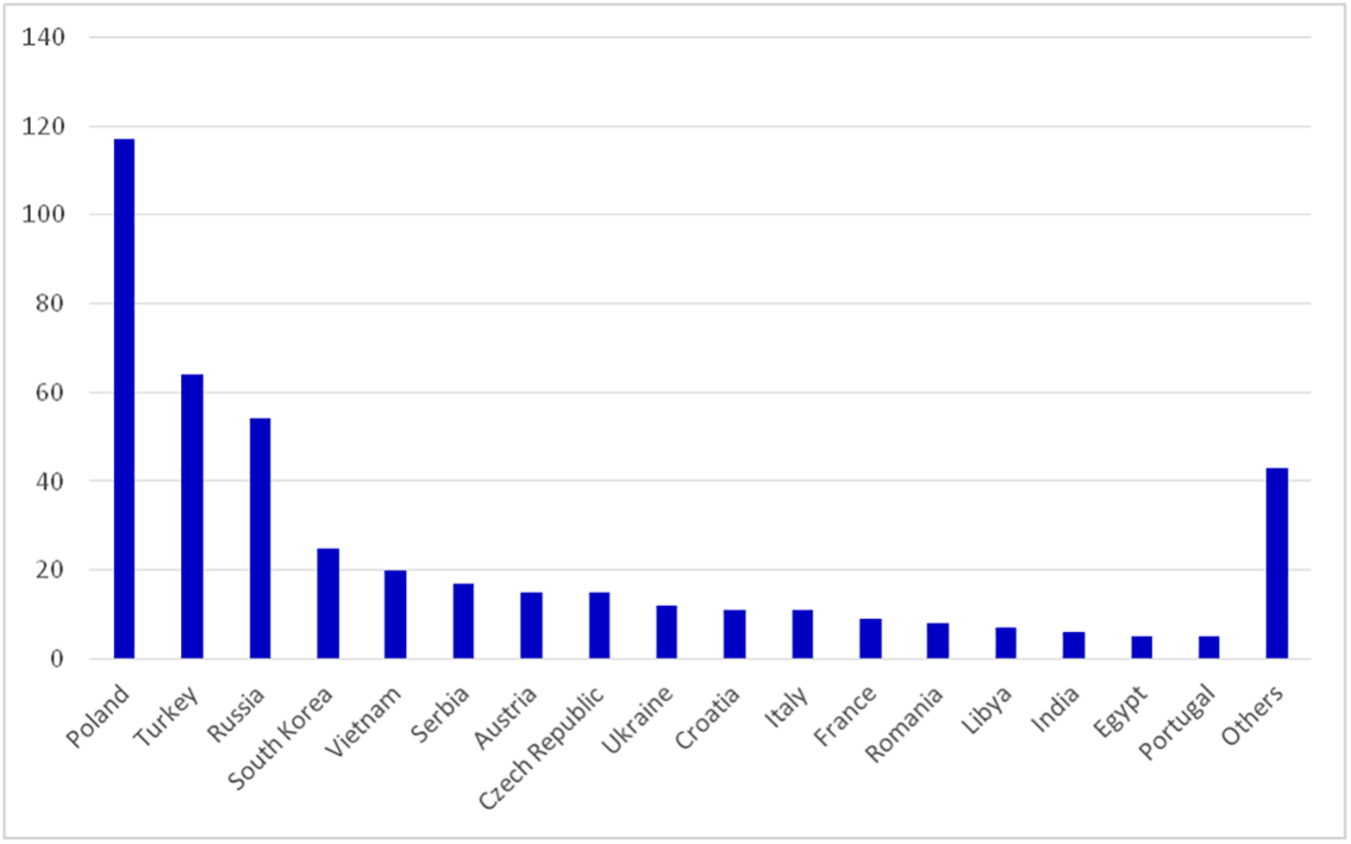
Numbers of immigrants in the study population according to land of origin.

The origins of the other immigrants are shown in Fig 2.

There were statistically significant differences in the comparison of Turkish immigrants in the general population to those in the study sample, where they were few. These differences occurred in the total group of Turkish immigrants and in the age groups 51–60, 61–70 and 71–90. In the younger and older age groups, the sample size is too small for valid statistical testing (n = 2 in the age group 18–30). There were significant differences in the age group 71–90 for Polish and Russian Immigrants, in which both ethnic groups are overrepresented.

Stratified by the three kinds of institutions, the proportion of immigrants is statistically significant in underrepresentation compared to the general Berlin population in all institutions: outpatient hospice services OR 0.33 (95%CI 0.28–0.39; p<0.0001), inpatient hospices OR 0.28 (95%CI 0.23–0.36; p<0.0001), palliative care wards OR 0.52 (95%CI 0.44–0.61; p<0.0001).

Considering the missing data (89 indeterminate questionnaires) as immigrant defined, the portion of the entire study sample would still be significant lower than in the general Berlin population (OR 0,43; 95% CI 0,39–0,47; p<0,0001).

## Discussion

This study provides a database of information on end-of-life care for immigrants for the first time in Germany. So far, this topic has only been researched qualitatively; while a look into the actual utilization of services have been lacking thus far. This investigation also shows a large study on immigration status with a simple survey instrument is possible.

The study was carried out in Berlin, a large city with a high proportion of immigrants and a dense network of HPC institutions. The results are therefore not easily transferable to other German federal states or regions, but they do indeed reflect the picture of other large German cities. The high response rate of 87% implies representativeness, yet not all hospice services participated in the data collection.

The descriptive evaluation shows that only 11.4% of the patients in the Berlin HPC institutions have an immigration background. The comparison of the entire study sample with the Berlin population shows that reduced representation of immigrants in the HPC institutions is statistically significant (p<0.0001).

Even though immigrants in Berlin are on average younger than native Germans, and reduced representation might be anticipated, the data show a significant underrepresentation in the largest age groups of the study population, age groups 51–60 and 61–70. These results are consistent with analyses from the field of nursing care. Schenk explains the phenomena of underrepresentation of immigrants in the nursing institutions by noting that the healthcare system caters toward the needs of the majority population and its offerings are oriented mainly toward the middle class [19], to which immigrants rarely belong.

Research from the United Kingdom likewise recognizes a low utilization of the HPC offerings by immigrants [20]. Presumed reasons for these findings according to Evans et al. are lack of awareness among immigrants about the offerings, lower referral rates of them, and deficient cultural sensibility among the providers. In addition the authors report a lower average age in the immigrant population and a lower rate of cancer.

Lack of cultural sensitivity might provide an explanation in the context of Germany: In the 10^th^ report of the Federal Government Committee for Immigration, Refugees, and Integration about the situation of foreigners in Germany and also in an expert report from the Robert Koch Institute on the 2^nd^ Poverty and Wealth Report of the Federal Government, deficits were emphasized in education and advanced training in regards to the intercultural domain and lack of culturally sensitive offerings in the hospitals, nursing care, and HPC [21]. Furthermore, the 18^th^ Research Report of the Federal Office for Migration and Refugees pointed out the immigrants’ lack of knowledge about the structures of medical care [3].

### Differences between the types of institutions

Of the types of institutions, immigrants were more likely to use palliative care wards at 15.6%; 10.6% used outpatient hospice services and 9.7% inpatient hospices. This is consistent with estimates by Tezcan-Güntekin and Razum who quantified the portion of immigrants in nursing institutions at 8.2% [22], if one takes into consideration that a majority of the assistance of outpatient hospice service takes place in nursing homes.

The higher utilization of the palliative care wards by immigrants could be related to the low-threshold offering: the route there via acute care hospitals, to which the palliative care wards are connected, is as a general rule a mere transfer from a normal ward within the same hospital, whereas the connection to an outpatient or inpatient hospice service requires, as a general rule, several schedulings and involvement from, for example, family, social services, and perhaps translators. Furthermore, the connection to a hospice service means also a decision for professional terminal care, while treatment on a palliative care ward can also follow other treatment goals, such as focused symptom control. Nevertheless, this clear difference should be the occasion to perceive the palliative care ward as a place in which end-of-life care can take place cross-culturally, is accepted, and thus also to clear the way for immigrants to further end-of-life care in hospices.

#### Turkish immigrants are rarely seen

The evaluation of the individual ethnic groups enables large differences to be recognized. Although persons with a Turkish immigration background form the largest immigrant group in Berlin, the rate of receiving end-of-life care in a Berlin HPC institution is only 0.23 compared to the whole Berlin population. Thereby, younger Turkish immigrants occur overall more frequently in the institutions than older Turks. Actually, a higher proportion should be expected, taking into consideration that most of the older Turkish immigrants did not returned to their homelands as initially planned due to better medical care in Germany and other reasons like having their grandchildren in Germany and financial constraints that hinders re-migration [23,24,25]. An explanation for these findings are given by Graaf et al. They conclude that Turkish and Moroccan immigrants “prefer to be cared for informally at home, and be able to support the strain related to that” [11]. Our findings support this conclusion as the number of Turkish patients is considerably low. Potential reasons might be, as already mentioned, language barriers and less access to HPC services [11,13]. But also, many Turkish immigrants are considered to spent many months a year in their country of origin [26], thus the resulting lower number of Turks in Berlin needs to be taken into account when interpreting the findings.

#### Lack of a “healthy immigrant” effect

Seventy-three percent of all Turkish immigrants in the study population were between the ages of 51 and 80 and thus altogether younger than non-immigrants in the HPC. Two studies–“Effects of the Nursing Future Development Bill” [27] and the “Twelfth Research Report: Nursing Needs and Demand for Nursing Services from Immigrants in Demographic Change” [28]–have already shown that immigrants in need of nursing care are on average 10 years younger than non-immigrants needing nursing. Furthermore, it is noteworthy that the proportion of nursing insurance services of the highest level (3) is 15% among immigrants compared to 9% among non-immigrants [28]. The authors of those studies reasoned that immigrants overall are less healthy, due to socioecomomic status including more difficult living conditions and less utilization of preventive services. The present study is able to support those results, since the immigrants were younger than non-immigrants including in the end-of-life care institutions. These results are surprising insofar as they contradict the so-called “healthy immigrant effect”. That asserts that (through self-selection or other-selection) above all healthy people constitute the labor immigrants [9]. If the “healthy immigrant effect” was the explanation for some time as to why immigrants have low uptake of health services, then one must postulate that it apparently faded away over time due to the extra strains of immigration (disconnection from one’s familiar environment, linguistic isolation, lower socioeconomic status), if in fact it ever really existed [29].

#### Polish and Russian immigrants are well represented

Polish immigrants are the largest immigrant group in all age groups in all three types of HPC institutions in Berlin. From age 61 onward, the portion of Polish immigrants in the HPC institutions is greater than in the Berlin population, though this difference is statistical significance only from age 71 onward. Also, Russian immigrants are overrepresented in all three types of HPC institutions in Berlin. From age 71 onwards they are present in the institutions more often than corresponds to their proportion in the Berlin population.

In the younger age groups, their proportion corresponds to that in the Berlin population. The calculations of the odds ratios show that among these three immigrant groups, Polish immigrants have the greatest chance to access HPC institutions in Berlin, and Turkish immigrants are least likely. If the OR was used as a measure of integration, the calculations correspond to the results of the Institute of Population and Development. According to them, both persons with Polish or Russian immigration backgrounds have good integration levels [30]. In contrast, integration was lowest for Turkish immigrants, above all in educational and socioeconomic areas. Presumably this also plays a role in regards to access to institutional care at the end of life.

How can the underrepresentation of Turkish immigrants be explained? Language barriers and lack of knowledge of the hospice and palliative offerings are potential explanations according to a survey of hospice staff [6]. But also refusal by the provider, out of worry about “difficult cases” played a decisive role in this survey.

In the qualitative part of the present research project, further possible explanations were found for the low utilization: loneliness in the institutions, food experienced as “foreign” and inadequate, other experiences of pain, lack of networking of the HPC providers with the local cultural and religious communities [16]. Although these results were gathered with a focus on East Asian immigrants, they show the acceptance of hospice offerings when they are carried out in a culturally sensitive way.

#### The refugee situation not yet considered

The present study does not reflect the current data on refugees and asylum-seekers in the Berlin population. Refugees with toleration status or in the asylum process are not excluded from the HPC, but they would hardly be able to orient themselves within the offerings of the German healthcare system. Ignorance often prevails on the part of the HPC, since asylum-seekers are often incorrectly assumed to only have a right to receive “emergency care”. Thus they may be viewed as even more underserved than the immigrants who have already been living in Germany for many years or decades [31].

Due to the current inflow of refugees (and regardless of some portion of them returning home later), many new first-generation immigrants will in the coming years live permanently in Germany and also die here. Therefore, the current refugee situation underscores the growing importance of the topic and contradicts the thinking that barriers to access will reduce themselves naturally in the course of time with second and third generation immigrants.

## Conclusions

Immigrants are underrepresented in the HPC institutions of Berlin. Despite this, a need for services exists as immigrants on average make use of care younger than non-immigrants. In this regard, the Turkish immigrants in particular are hardly found, despite being the largest immigrant population in Germany. These results should prompt research on Turkish immigrants, in regards to barriers to access. In the comparison of the kinds of institutions, one sees a decline between the palliative care wards and the outpatient hospice services, in which immigrants hardly access. Apparently, the palliative care wards appear to be a smaller hurdle for utilization, which could give them the character of a “door opener” to HPC for immigrants. The high uptake of the only intercultural hospice service in Berlin shows the acceptance of this form of care by immigrants. The data presented here should also give rise to an urgency of action, for it shows a non-existent, or non-demonstrable, “healthy immigrant effect” of the first generation of work immigrants who are now entering old age. Active dialogue with the cultural and religious communities that immigrants represent, is a key role in the efforts to provide adequate and fair access to end-of-life care for all people in Berlin.
